# Supplementary material for: Transposons contribute to the acquisition of cell type-specific cis-elements in the brain
Source: Commun Biol. 2023 Jun 10;6:631. doi: 10.1038/s42003-023-04989-7 (PMC10257727; doi:10.1038/s42003-023-04989-7)
Supplement: Supplementary file 2 — Supplementary Information [file 42003_2023_4989_MOESM2_ESM.pdf]

Supplementary Information

Transposons contribute to the acquisition of cell type-specific cis-elements in the brain

Kotaro Sekine [1,2], Masahiro Onoguchi [1,2*] and Michiaki Hamada [1,2,3*]

[1]Graduate School of Advanced Science and Engineering, Waseda University, Tokyo, Japan.
[2]Computational Bio Big-Data Open Innovation Laboratory (CBBD-OIL), National Institute of Advanced Industrial Science and Technology (AIST), Tokyo, Japan.
[3]Graduate School of Medicine, Nippon Medical School, Tokyo, Japan.

[*]To whom correspondence should be addressed. Tel: +81 3 5286 3130; Fax: +81 3 5286 3130; Email: m.onoguchi@aoni.waseda.jp (MO) and mhamada@waseda.jp (MH).

Supplementary Data

Supplementary Data 1. The percentage of ATAC peaks that overlap with TEs for each cell belonging to the cell type (described in Figure 4a,b). Each row shows the cell names, the number of ATAC peaks, the number of ATAC peaks that overlap with TEs, the percentage of ATAC peaks overlapping with TEs, and the label (celltype, pseudo-time branch) of cell to which it belongs.
Supplementary Data 2. Distribution of classes of the TE that resides in transcription factor binding sites or ATAC-seq peaks (described in Figure 4c,d). (a) Each row shows the position of overlap between ChIP-seq peaks and TEs, and TE class of the TEs to which it belongs. (b) Each row shows the position of ATAC-seq peaks which overlap with TE-derived *de novo* motifs, and TE class of the TEs to which it belongs.
Supplementary Data 3. Enrichment scores of ChIP peaks or accessible motifs for TEs (described in Figure 5a). (a,b) Each row shows the type (TE class, family) of TE subfamily to which it belongs, the ChIP peak enrichment score (ES_peak), the accessible motif enrichment score (ES_motif), and Fisher's exact test (p-value) with the Benjamini–Hochberg correction (q-value) based on the numbers used to calculate the enrichment scores of ES_peak or ES_motif. (c,d) Each row shows the type (TE class, family) of TE subfamily to which it belongs, and robust z-score using the accessible motif enrichment score (ES_motif) based on the chromVAR's background peak set as a control.
Supplementary Data 4. Two-group comparison of bias corrected accessibility deviations of TE subfamilies between branch (S2, S1) and branch (S0, S1) (described in Figure 5d). Each row shows the difference between the mean TE deviation score of branch (S2, S1) and the mean TE deviation score of branch (S0, S1), p-value using the Mann–Whitney U test, and q-value with Benjamini–Hochberg correction.

Visualization of known labels for cells in the two-dimensional map generated with t-SNE based on 7-mer chromatin accessibility

Supplementary Figure 1 shows the distribution of known labels for the cells in the two-dimensional map generated with t-SNE based on 7-mer chromatin accessibility in Figure 2a.

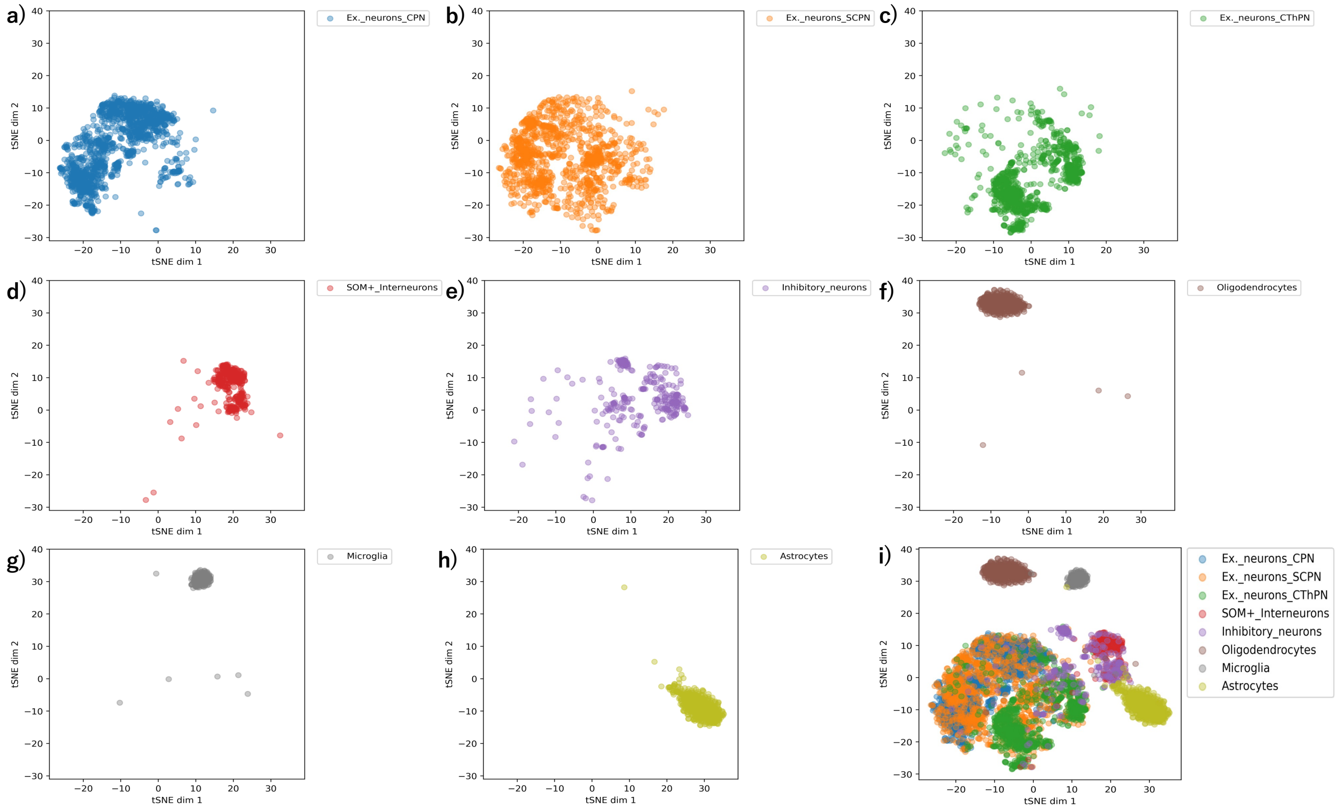

Supplementary Figure 1. Visualization of cell-type labels for cells in the two-dimensional map generated with t-SNE based on 7-mer chromatin accessibility. Each figure shows the distribution of cells with the corresponding known cell-type labels defined by Cusanovich *et al.* [1] on the two-dimensional map generated with t-SNE. The distributions for each label are shown in (a)–(h), and the distributions for all labels are shown in (i). (a) shows Ex._neurons_CPN-labeled cells (1391 cells), (b) shows Ex._neurons_SCPN-labeled cells (1160 cells), (c) shows Ex._neurons_CThPN-labeled cells (995 cells), (d) shows SOM+_Interneurons-labeled cells (386 cells), (e) shows Inhibitory_neurons-labeled cells (320 cells), (f) shows Oligodendrocyte-labeled cells (458 cells), (g)shows Microglia-labeled cells (197 cells), and (h) shows Astrocyte-labeled cells (551 cells).

Visualization of the accessibility of the seed k-mer used to generate the *de novo* motif for each cell-type.

Supplementary Figure 2 shows the accessibility of the seed k-mer used to generate the *de novo* motif for each cell-type.

a) *de novo* 1 (CAGATGG)

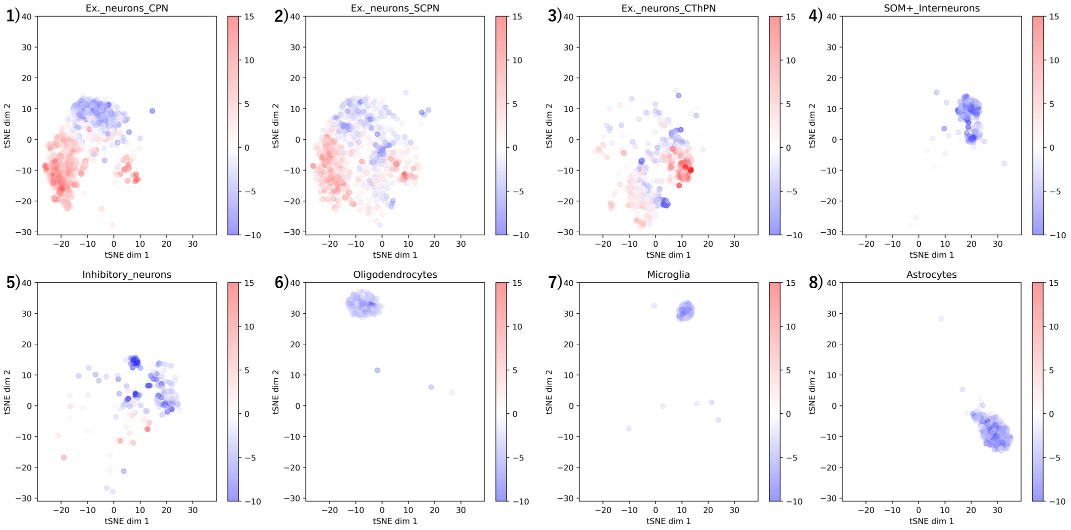

b) *de novo* 2 (CACCCAC)

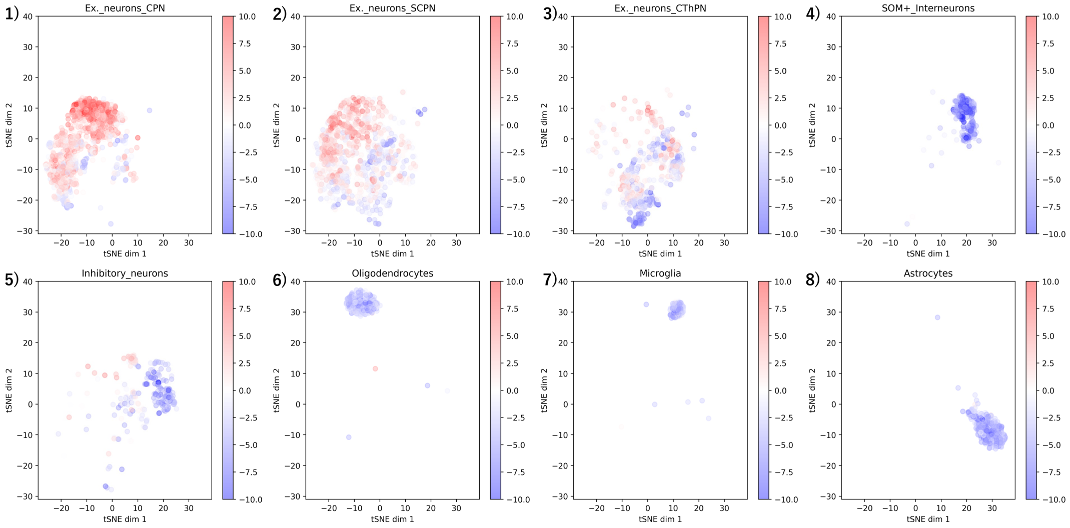

c) *de novo* 3 (CTAATTA)

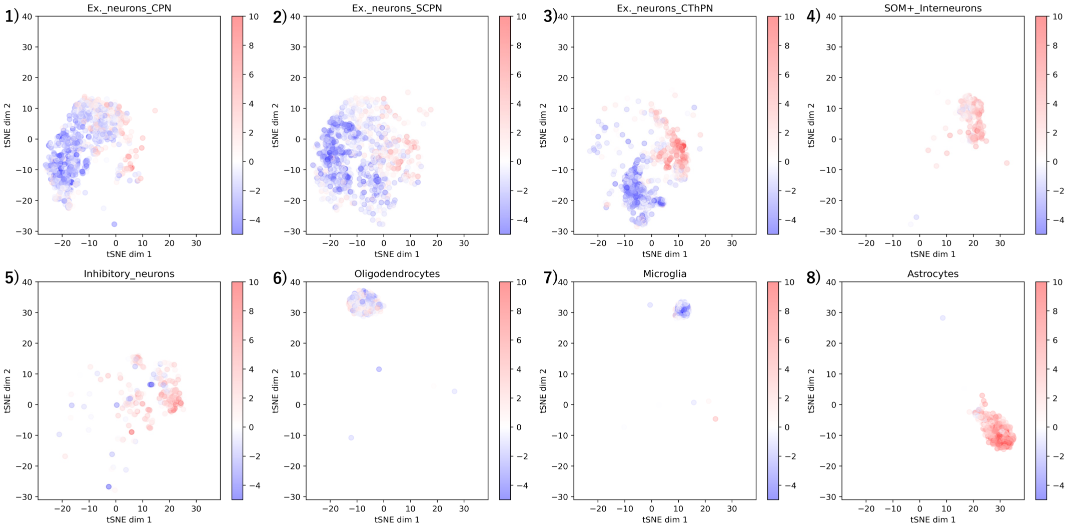

d) *de novo* 4 (ACAGCTG)

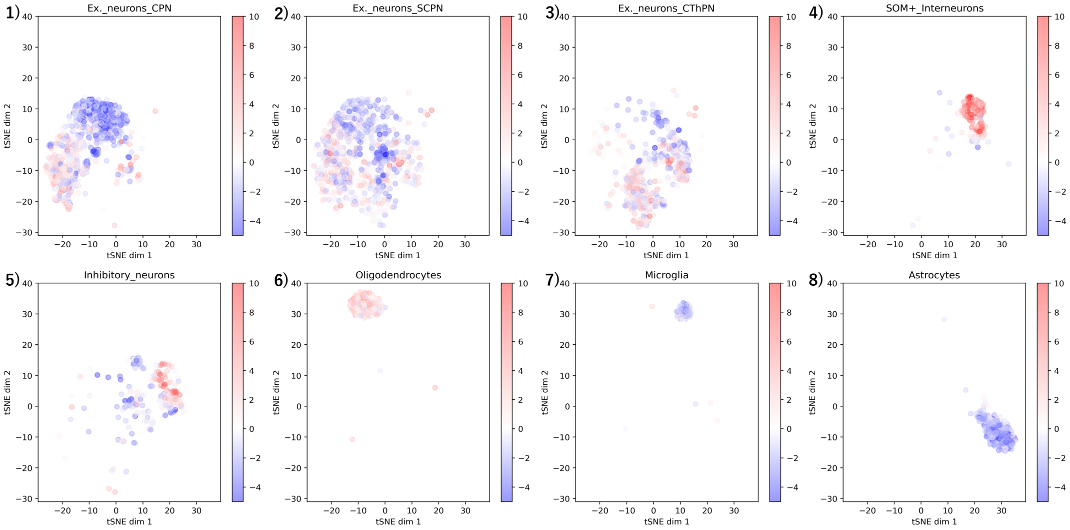

Supplementary Figure 2. Visualization of the accessibility of the seed k-mer of the *de novo* motifs on the two-dimensional map generated with t-SNE for each cell-type. The panels a), b), c), and d) show *de novo* motifs 1, 2, 3, and 4, respectively. In each panel, (1) shows Ex._neurons_CPN-labeled cells, (2) shows Ex._neurons_SCPN-labeled cells, (3) shows Ex._neurons_CThPN-labeled cells, (4) shows SOM+_Interneurons-labeled cells, (5) shows Inhibitory_neurons-labeled cells, (6) shows Oligodendrocyte-labeled cells, (7) shows Microglia-labeled cells, and (8) shows Astrocyte-labeled cells. The cell-type labels were defined by Cusanovich *et al.* [1]. Each cell is colored by deviation z-scores (red: high, blue: low).

Known motifs similar to the *de novo* motifs

Supplementary Figure 3 shows the known motifs (provided by HOCOMOCO v11 database [2]) which are similar to *de novo* motif 1, *de novo* motif 2, *de novo* motif 3, and *de novo* motif 4.

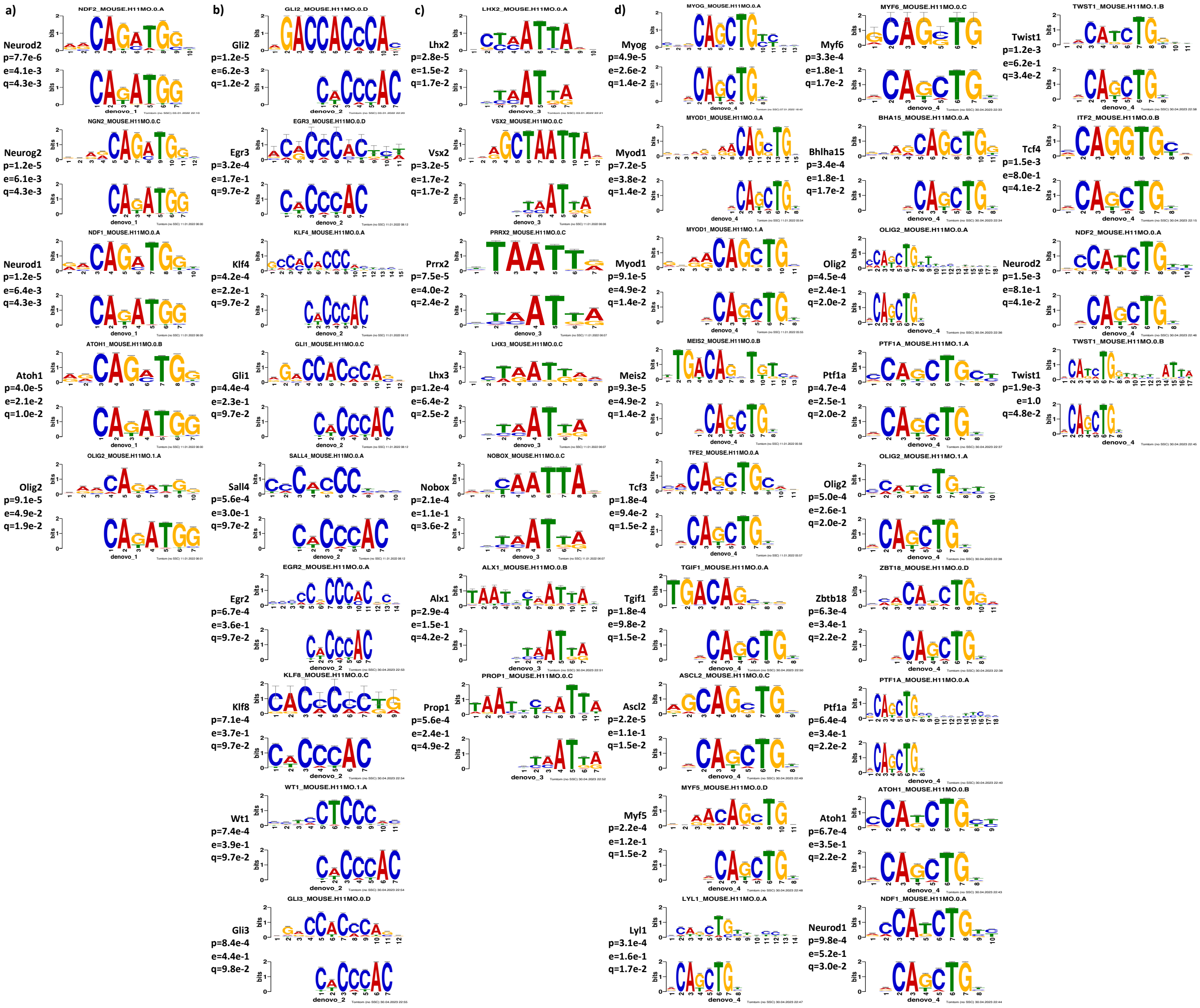

Supplementary Figure 3. The top five known motifs that are significantly similar to each *de novo* motif. Each panel shows the results of the TOMTOM [3] comparison between known motifs and *de novo* motifs. (a) Motifs similar to *de novo* motif 1 ($q < 0.05$). (b) Motifs similar to *de novo* motif 2 ($q < 0.1$). (c) Motifs similar to *de novo* motif 3 ($q < 0.05$). (d) Motifs similar to *de novo* motif 4 ($q < 0.05$).

Gene activity of the candidate transcription factors for each *de novo* motif

Supplementary Figure 4 shows the gene activity levels of the candidate transcription factors with motifs similar to *de novo* motifs in the cell-types corresponding to Figure 2a (the detailed methods of calculating gene activity levels are presented in "Data sources".

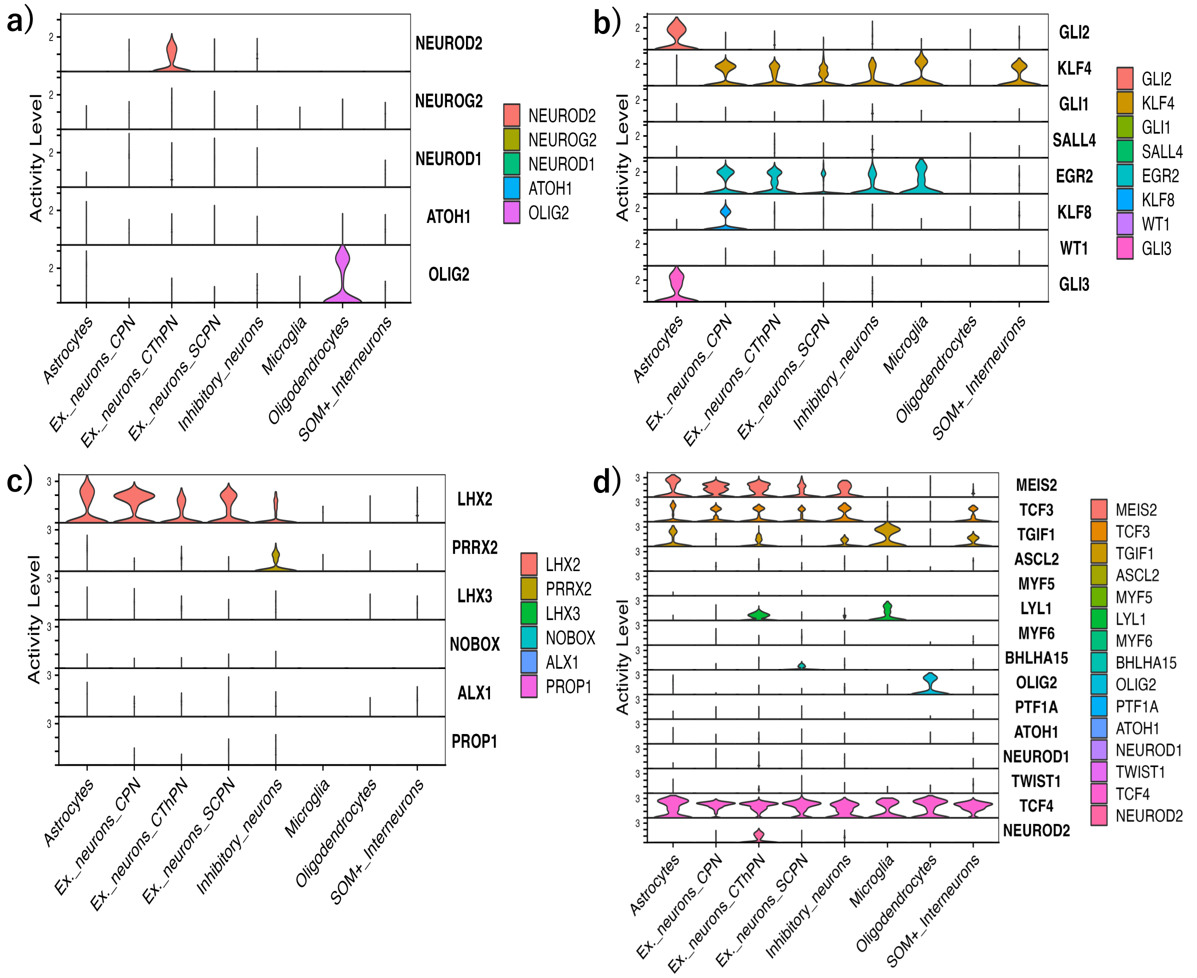

Supplementary Figure 4. Gene activity levels of the candidate transcription factors whose binding motifs were similar to *de novo* motifs 1–4. Each panel shows the gene activity levels calculated with scATAC-seq data (a: *de novo* motif 1, b: *de novo* motif 2, c: *de novo* motif 3, d: *de novo* motif 4). The color of the plot shows $\log_{10}$(gene activity + 1).

Known labels for cells belonging to each branch

Supplementary Figure 5 shows the distribution of known labels for the cells belonging to each branch as a result of the pseudo-time analysis with STREAM [4] in Figure 3a.

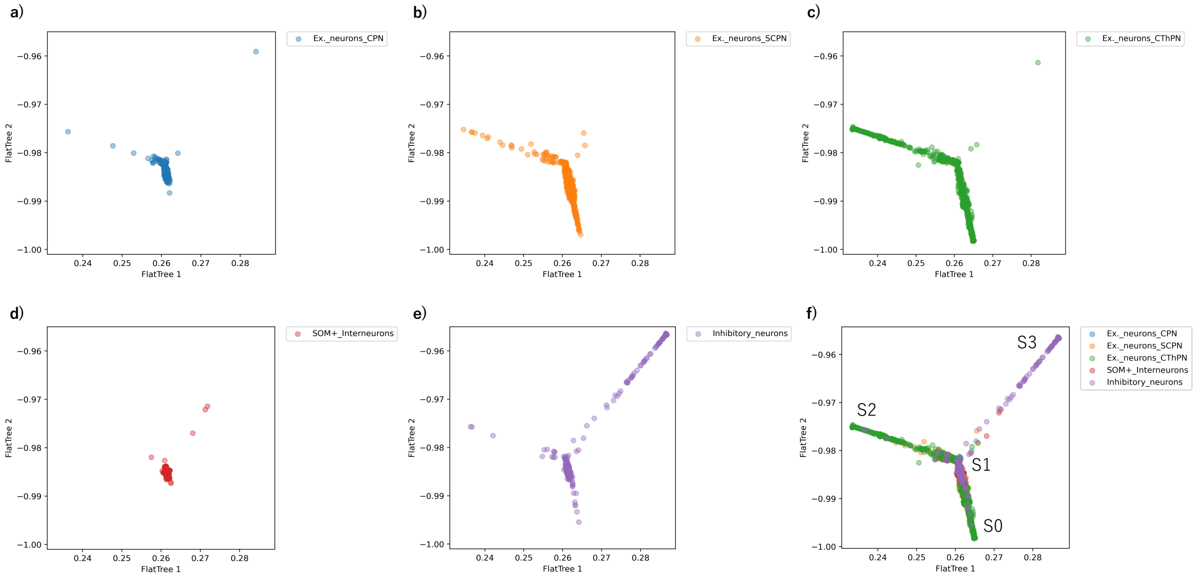

Supplementary Figure 5. Composition of cell type labels for cells in each branch. Each figure shows the distribution of cells with the corresponding known cell type labels defined by Cusanovich *et al.* [1] in the branch. The distributions for each label are shown in (a)–(e), and the distributions for all labels are shown in (f). (a) Number of Ex._neurons_CPN-labeled cells in branch (S0, S1) was 1262, in branch (S2, S1) was 81, and in branch (S3, S1) was 48. (b) Number of Ex._neurons_SCPN-labeled cells in branch (S0, S1) was 1097, in branch (S2, S1) was 55, and in branch (S3, S1) was 8. (c) Number of Ex._neurons_CThPN-labeled cells in branch (S0, S1) was 712, in branch (S2, S1) was 278, and in branch (S3, S1) was 5. (d) Number of Inhibitory_neurons-labeled cells in branch (S0, S1) was 225, in branch (S2, S1) was 14, and in branch (S3, S1) was 81. (e) Number of SOM+_Interneurons-labeled cells in branch (S0, S1) was 382, in branch (S2, S1) was 1, and in branch (S3, S1) was 3.

Visualization of the accessibility of the seed k-mer used to generate the *de novo* motif for cells belonging to each branch.

Supplementary Figure 6 shows the accessibility of the seed k-mer used to generate the *de novo* motif for the cells belonging to each branch as a result of the pseudo-time analysis with STREAM [4] in Figure 3a.

a) *de novo* 1 (CAGATGG)

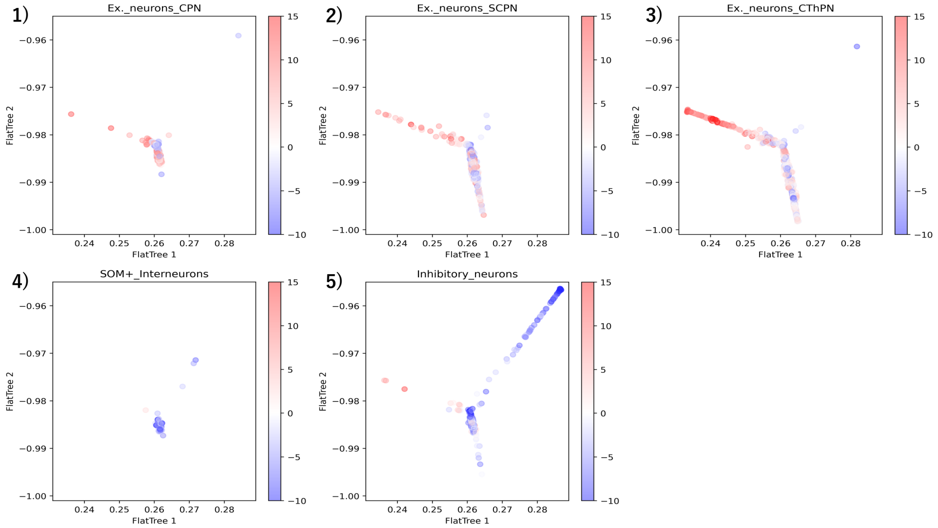

b) *de novo* 2 (CACCCAC)

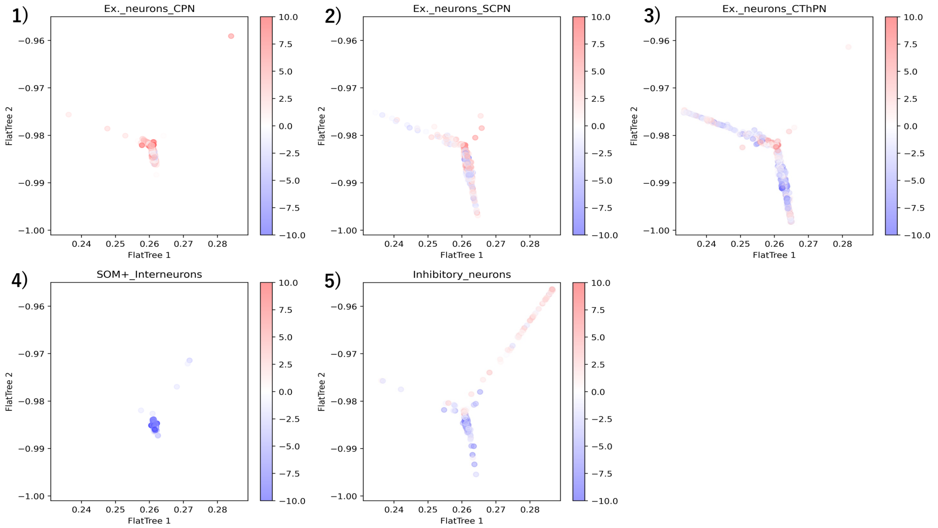

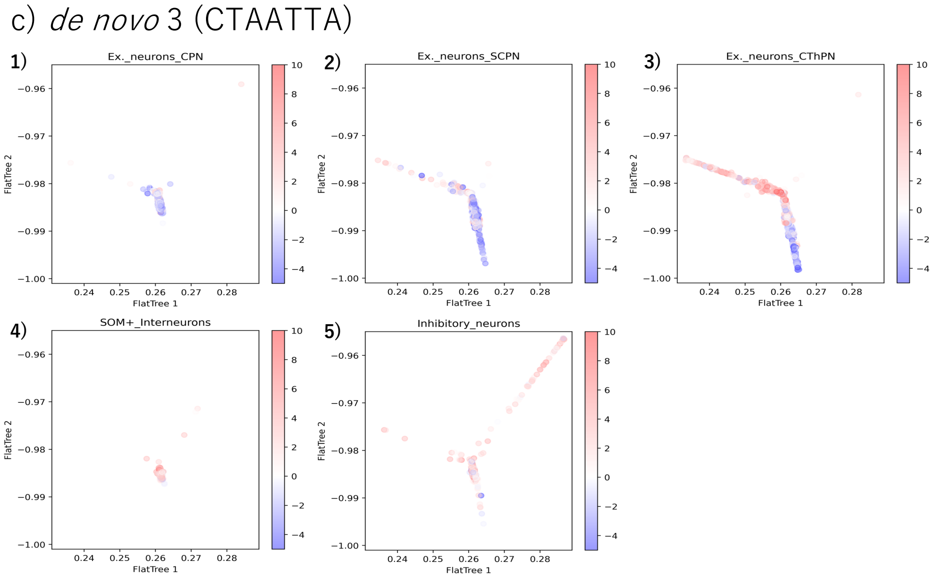

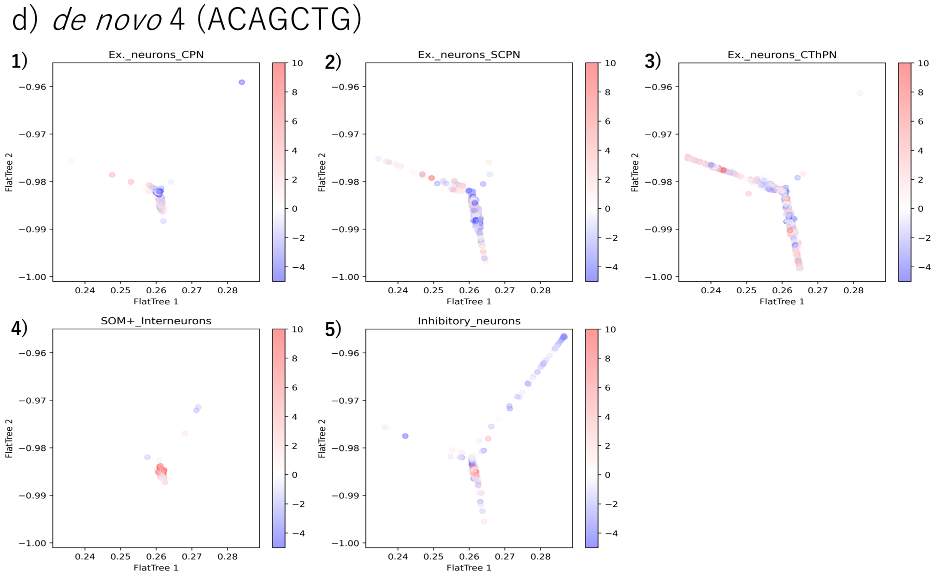

Supplementary Figure 6. Visualization of the accessibility of the seed k-mer of the *de novo* motifs on the pseudo-time branch generated with STREAM for each cell type. The panels a), b), c), and d) show *de novo* motifs 1, 2, 3, and 4, respectively. In each panel, (1) shows Ex._neurons_CPN-labeled cells, (2) shows Ex._neurons_SCPN-labeled cells, (3) shows Ex._neurons_CThPN-labeled cells, (4) shows SOM+_Interneurons-labeled cells, and (5) shows Inhibitory_neurons-labeled cells, respectively. The cell-type labels were defined by Cusanovich *et al.* [1]. Each cell is colored by deviation z-scores (red: high, blue: low).

Motif accessibility and gene activity of differentiation status marker genes in each branch

Supplementary Figure 7a,b shows the distributions of motif accessibility / gene activity up to the node S2 (glutamatergic neuronal progenitor cells) and the node S0 (mature neurons), using the results of pseudo-time analysis with STREAM [4] in Figure 3b. We used the following marker genes: neuronal progenitor cells (*Neurog2*, *Lhx2*, *Neurod1*, (*Neurod2*)), maturing neurons (*Neurod2*, *Mef2c*) [5–9]. In Supplementary Figure 7a, we show changes in motif accessibility along the pseudo-time using the deviation z-scores of known transcription factor motifs calculated with chromVAR [10]. In Supplementary Figure 7b, we show the changes in gene activity along the pseudo-time using the gene activity scores provided by Cusanovich *et al.* [1]. The results showed high activation of marker genes for neuronal progenitors in branch (S2, S1) and mature neurons in branch (S0, S1). In Supplementary Figure 7c, we show the high gene activities of *Dlx6* and *Gad2* in branch (S3, S1), which are associated with the function of GABAergic neurons [11].

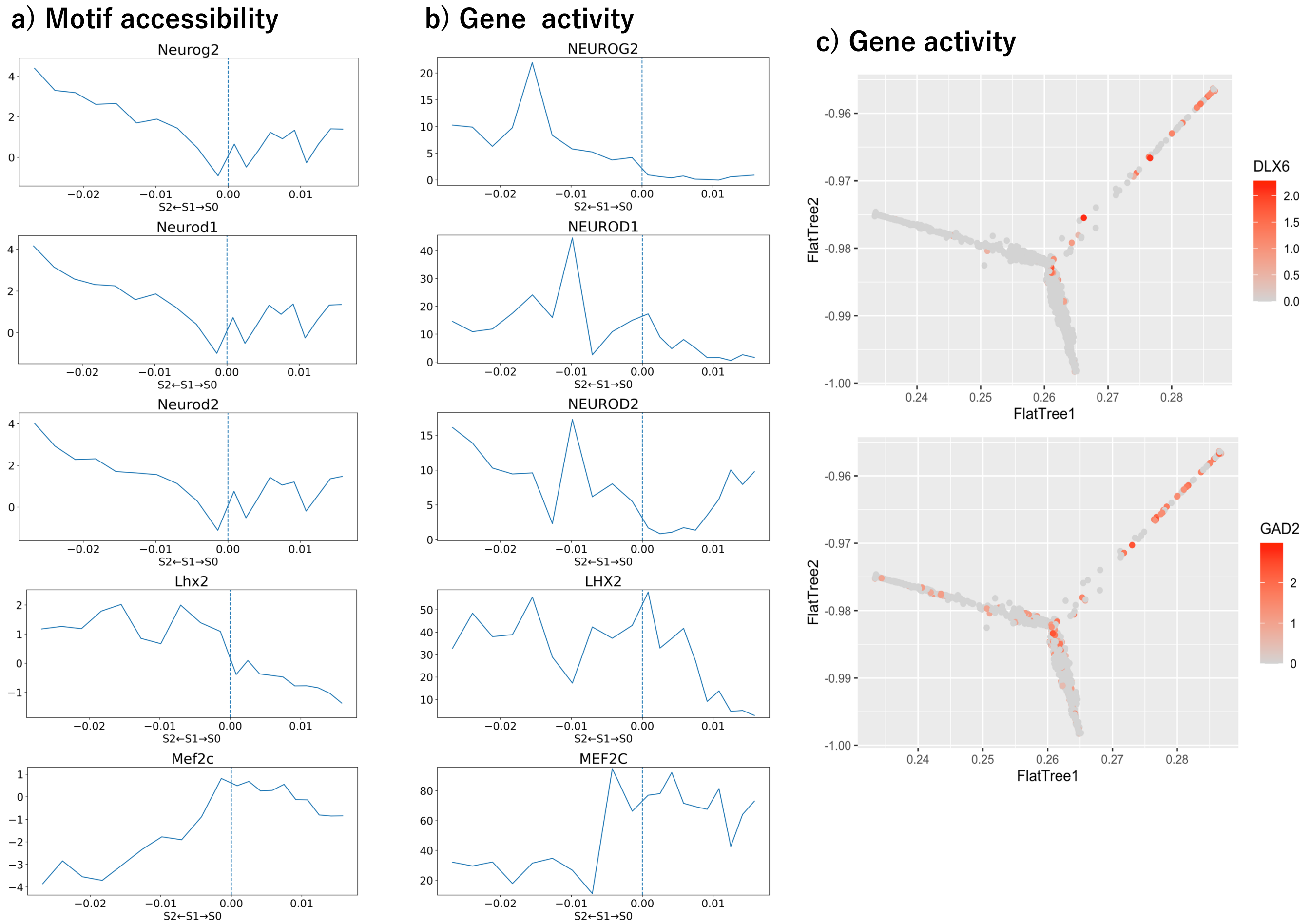

Supplementary Figure 7. Marker genes of neuronal progenitor cells are activated in branch (S2, S1), and mature neurons are activated in branch (S0, S1). (a, b) The marker gene activity is compared between the (S2, S1) and the (S0, S1) branches based on the distribution of (a) motif accessibility or (b) gene activity scores. The graph shows the (a) average motif accessibility or (b) gene activity score of key lineage genes for each segment when the pseudo-time of each branch is equally divided into 10 segments. The x-axis shows the pseudo-time from the branching point S1 (0) to the nodes S2 (-0.027) or S0 (0.016). The y-axis shows the average of (a) motif deviation z-score or (b) gene activity score for each segment. (c) The activity of Dlx6 and Gad2 genes (function in GABAergic neurons) in each cell was mapped to the branch. The color of the plot shows $\log_{10}(\text{gene activity} + 1)$.

GO analysis of differentially activated genes between the branches

Supplementary Figure 8 shows the GO terms enriched in the list of genes whose activities are different ($\log_2$ fold > 2; $q < 0.05$: Mann–Whitney U test with Benjamini–Hochberg correction) between branch (S2, S1) and branch (S0, S1). In Supplementary Figure 8, the GO terms that are enriched ($q < 0.05$) in the list of highly active genes in branch (S2, S1) and branch (S0, S1) are shown as terms that are commonly enriched at the top or terms that are enriched at the top in only one of them.

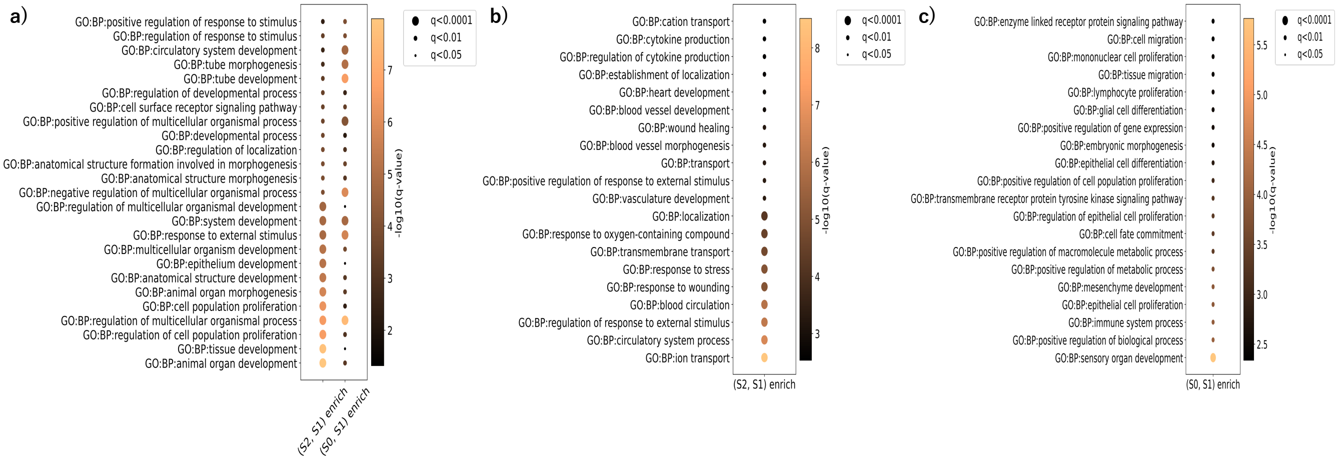

Supplementary Figure 8. Neuronal progenitor cell-labeled branch (S2, S1) is enriched ($q < 0.05$) for genes with functions that may be involved in a stress-dependent manner and involved in tissue repair. The results show the GO analysis (biological process) of the list of genes whose activity is different between the branch (S2, S1) and the branch (S0, S1). The color depth of the plots indicates the enrichment scores in $-\log_{10}$ (q-value), and the size was determined according to (q-value). In the panel (a), terms commonly enriched in both branch (S2, S1) and branch (S0, S1) and are included in the top 20 terms in branch (S2, S1) or branch (S0, S1) are shown. In the panel (b), the top 20 terms enriched only in branch (S2, S1) are shown. In the panel (c), the top 20 terms enriched only in branch (S0, S1) are shown.

Characteristics of TE-derived cis-elements for each cell-type

Supplementary Table 1 shows the degree of overlap between the TE and pseudo-bulk ATAC peaks for each cell-type label using randomly sampled cells (the detailed methods are presented in "Comparison of TE-ATAC peak overlap between cell-types using randomly sampled cells").

Supplementary Table 1. The degree of overlap between the TE and pseudo-bulk ATAC peaks for each cell-type. Each cell indicates the number of bulk ATAC peaks that overlap or do not overlap with TE using randomly sampled cells from each cell-type. Each row indicates the cell-type of interest for random sampling, and each column indicates whether the ATAC peaks overlap or do not overlap with the TE.

	TE	nonTE
Ex._neurons_CPN	14169	30072
Ex._neurons_SCPN	7094	17951
Ex._neurons_CThPN	8617	21841
SOM+_Interneurons	5537	18905
Inhibitory_neurons	4155	15982
Microglia	3681	10536
Astrocytes	2283	10382
Oligodendrocytes	2051	8701

Histone modifications in TE-derived accessible motifs

Supplementary Table 2 shows the overlap of TE-derived accessible motifs with histone modification ChIP peaks. We showed that the ATAC peaks that intersect with each TE-derived *de novo* motif tended to overlap significantly ($p < 0.01$: Fisher's exact test) with the enhancer label (H3K4me1) compared to the promoter label (H3K4me3).

Supplementary Table 2. TE-derived accessible *de novo* motifs 1–3 tend to function as enhancers. Each number indicates the number of motif-containing bulk ATAC peaks (obtained in "Data sources") that overlap with the corresponding genomic regions. Each row indicates the TE and non-TE regions. The row that overlaps with TEs indicates cases in which motifs are also inside the TEs. For each column, + indicates that the histone modification peak is present, and − indicates that the histone modification peak is absent.

Motif	Condition	H3K4me1+/H3K4me3−	H3K4me1−/H3K4me3+
de novo 1	TEs	520	115
	Non-TE	3438	2030
de novo 2	TEs	553	121
	Non-TE	3342	3145
de novo 3	TEs	105	27
	Non-TE	786	610

Multiple alignments of TE fragments overlapping ATAC peaks

Supplementary Figure 9 shows the multiple alignments of TE fragment sequence sets that overlap ATAC peaks.

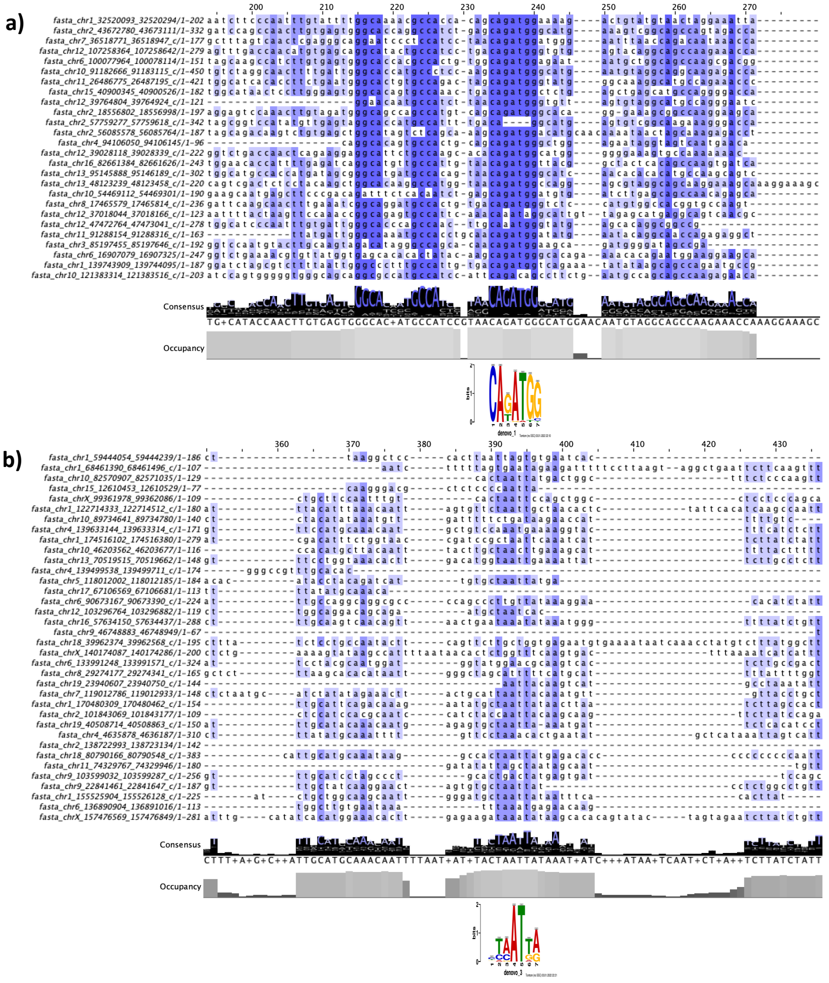

Supplementary Figure 9. Multiple alignments of the accessible genomic MER130 and MamRep434 fragments with or without the *de novo* motifs. The panels show the result of multiple alignments of (a) MER130 (n = 26) and (b) MamRep434 (n = 36) fragment sequences that overlap bulk ATAC peaks by at least 1 bp. Each panel shows ±40 bp of the detection site of the *de novo* motifs in the multiple alignments. The left column on each panel indicates the genomic position of each TE fragment, and the numbers above the alignments indicate arbitrary aligned positions. The shade of blue highlight shows the degree of the matched nucleotide with the aligned nucleotides in each position. The consensus annotation shows the representative nucleotide based on the proportion of aligned nucleotides in each position. The consensus sequence of the multiple alignments is indicated below the consensus annotation. The occupancy annotation shows the number of nucleotides aligned at each position in the multiple sequence alignments. The bottom motif logo shows the corresponding position of *de novo* motifs 1 and 3, respectively.

IP/input ratio of ChIP-seq reads at the sites of TE-derived *de novo* motifs

Supplementary Figure 10 shows the proportion of the IP and input reads to the total reads of Neurod2 (a) or Lhx2 and (b) ChIP-seq at the detection sites of MER130-derived *de novo* motif 1 (a) or MamRep434-derived *de novo* motif 3 (b), respectively. ChIP-seq IP reads were more concentrated than input reads in the detection sites of *de novo* motifs in MER130 and MamRep434.

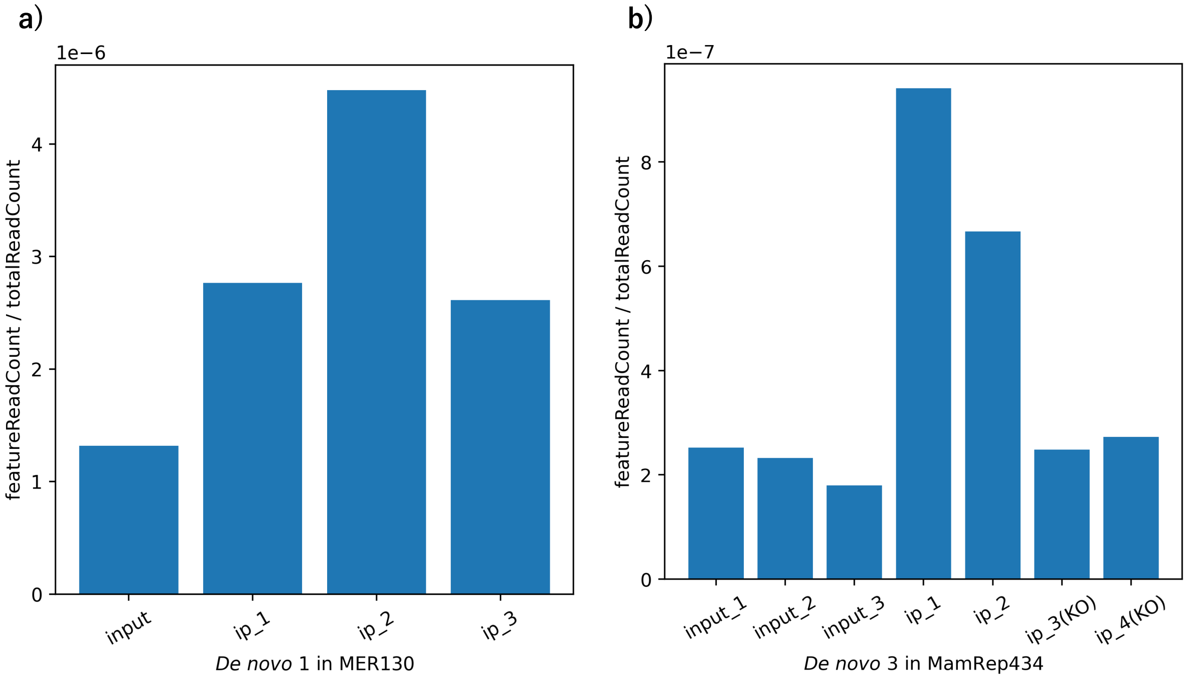

Supplementary Figure 10. Comparison between IP and input ChIP-seq reads on the *de novo* motifs in the TEs. (a) Neurod2 ChIP-seq data on the sites of the MER130-derived *de novo* motif 1. (b) Lhx2 ChIP-seq data on the sites of the MamRep434-derived *de novo* motif 3. In each panel, the y-axis shows the ratio of the number of IP or input reads to the total number of reads. The data were acquired from (a) input (ip): SRX1979972, ip_1: SRX1979968, ip_2: SRX1979969, ip_3: SRX1979970, (b)input_1: SRX2487350, input_2: SRX2487351, input_3: SRX3064887, ip_1: SRX2487346, ip_2: SRX2487347, ip_3(KO): SRX2487356, and ip_4(KO): SRX3064886.

Distribution of the annotated regions converted from genome to consensus TE sequences

Supplementary Figure 11 shows the distribution of the ChIP-seq peak regions and the detection sites of accessible *de novo* motifs converted from the genome to consensus TE sequences.

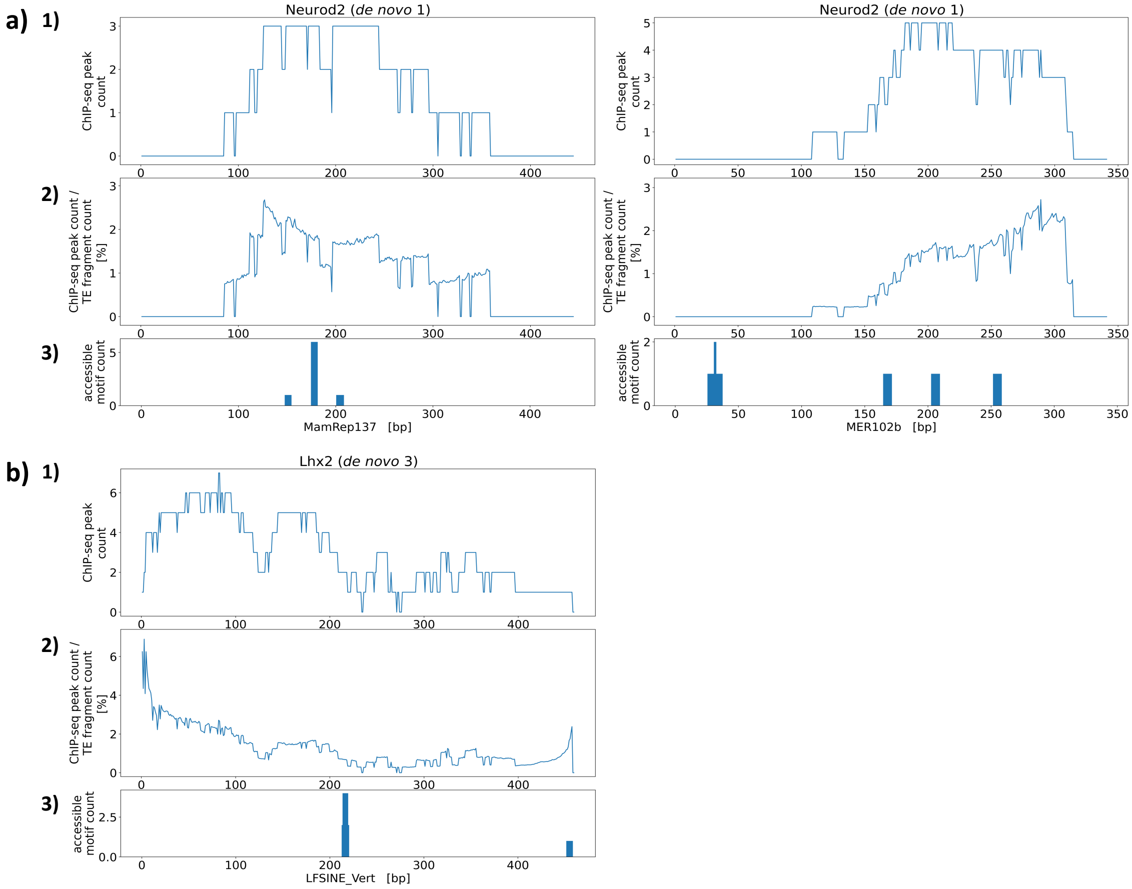

Supplementary Figure 11. Distribution of the transcription factor binding and accessible *de novo* motifs in the TE consensus sequences. (a) Distribution of Neurod2 binding and accessible *de novo* motif 1 in the TE consensus sequence (left: MamRep137, right: MER102b). (b) Distribution of the Lhx2 binding and accessible *de novo* motif 3 in the TE consensus sequence (LFSINE_Vert). In each panel, the y-axis shows 1) the number of ChIP-seq peaks mapped to the consensus TE sequences, 2) the percentage of ChIP-seq peaks normalized by the total number of TE fragments in the genome, and 3) the number of the detection sites of the accessible *de novo* motifs mapped from the genome to the consensus TE sequences. The x-axis shows the relative positions of each TE consensus sequence.

Neighboring genes of TE-derived accessible motifs

The lists of genes located in the vicinity of TE-derived accessible motifs with GREAT [12] are shown in Supplementary Table 3, Supplementary Table 4. Supplementary Table 3 shows the neighboring genes of accessible *de novo* motif 1 (Neurod2-like motif)-containing MER130 TEs, and Supplementary Table 4 shows the neighboring genes of accessible *de novo* motif 3 (Lhx2-like motif)-containing MamRep434 TEs.

Supplementary Table 3. Candidate target genes of MER130-derived accessible *de novo* 1 motifs. Each row shows the genomic loci of the bulk ATAC peaks (obtained in "Data sources" that overlap with MER130 TE with internal motifs, and the neighboring genes of the ATAC peaks obtained with GREAT [12]. The number next to the genes indicates the distance between the TSS of the gene and the corresponding ATAC peak.

ATAC peak region	Candidate target genes (distance to TSS [bp])
chr1:32520009-32520508	*Gm5415* (+83879), *Khdrbs2* (+290608)
chr1:139743856-139744355	*Nr5a2* (-887102), *Ptprc* (+327776)
chr2:18556456-18556955	*Dnajc1* (-242249), *Commd3* (-37344)
chr2:43672685-43673184	*Arhgap15* (+68545)
chr2:56085380-56085879	*Kcnj3* (+797249), *Nr4a2* (+881819)
chr3:85197374-85197873	*Gatb* (-180417), *Fbxw7* (+441556)
chr4:94105772-94106271	*Gm10306* (-117215)
chr6:16907020-16907519	*Tes* (-107879), *Tfec* (-58829)
chr6:100077618-100078117	*Prok2* (-401482), *Rybp* (+159567)
chr7:36518640-36519139	*Pdcd5* (-86350), *Dpy19l3* (+20583)
chr8:17465411-17465910	*Csmd1* (+69620)
chr10:54469002-54469501	*Man1a* (-673650)
chr10:91182670-91183169	*Tmpo* (-548593)
chr11:26486644-26487143	*Vrk2* (+4835), *Fancl* (+200759)
chr11:91288203-91288702	*Tom1l1* (-739532), *Kif2b* (+150470)
chr12:39028028-39028527	*Etv1* (-478391), *Dgkb* (+420986)
chr12:39764648-39765147	*Etv1* (+258229), *Arl4a* (+999118)
chr12:107258105-107258604	*Vrk1* (+9882)
chr13:48123216-48123715	*Id4* (-233330), *Rnf144b* (+905377)
chr13:95145730-95146229	*Tbca* (-412918), *Ap3b1* (+17065)
chr15:40900300-40900799	*Oxr1* (-378478), *Zfpm2* (+413962)
chr16:82661123-82661622	NONE

Supplementary Table 4. Candidate target genes of MamRep434-derived accessible *de novo* 3 motifs. Each row shows the genomic loci of the bulk ATAC peaks (obtained in "Data sources" that overlap with MamRep434 TE with internal motifs, and the neighboring genes of the ATAC peaks obtained with GREAT [12]. The number next to the genes indicates the distance between the TSS of the gene and the corresponding ATAC peak.

ATAC peak region	Candidate target genes (distance to TSS [bp])
chr1:59443936-59444435	*Fzd7*(-94805), *Cdk15* (+130436)
chr1:155525437-155525936	*Rgs16* (-61792), *Rgs8* (+25532)
chr1:170480155-170480654	*Pbx1* (-118377), *Nuf2* (+981190)
chr2:138722805-138723304	*Sptlc3* (-596601), *Btbd3* (+618826)
chr5:118011983-118012482	*Nos1* (-279920), *Ksr2* (+147395)
chr10:82570512-82571011	*Chst11* (+122519), *Slc41a2* (+229865)
chr18:80790051-80790550	*Ctdp1* (-123867), *Nfatc1* (+119509)
chr19:40508605-40509104	*Pdlim1* (-162749), *Sorbs1* (+79414)

The estimated acquisition time during evolution for TE-derived cis-elements accessible in each cell-type

Supplementary Figure 12 shows the estimated acquisition time during evolution for MER130- and MamRep434-derived cis-elements accessible in each cell-type or branch.

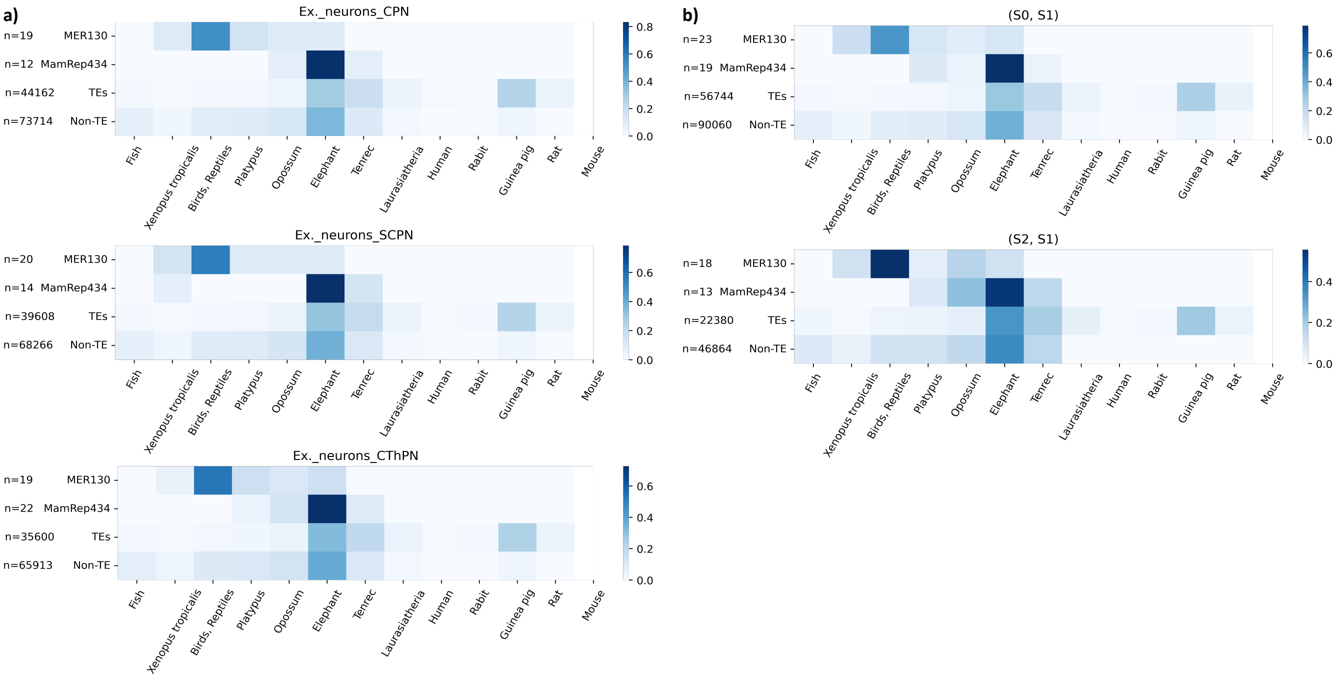

Supplementary Figure 12. MER130- and MamRep434-derived cis-elements were acquired mainly in the ancestors of Amniota and Eutheria, respectively, in excitatory neurons. (a, b) The estimated acquisition time during evolution for cis-elements derived from MER130, MamRep434, all TEs, or non-TEs and accessible in each cell-type (a) or branch (b). The heatmap shows the percentage of peaks assigned to a particular clade out of the total number of bulk ATAC peaks (n). Bulk ATAC peaks were obtained by calling peaks from scATAC-seq bam files split by each cell population and resizing the peak length to 500 bp, as shown in “Data sources”. The number of species used for analysis in each clade is indicated by ⟨⟩. The values on the branches are divergence times (in a million years ago, Mya).

Sequence conservation of TE-derived accessible motifs

Supplementary Figure 13 shows the phyloP conservation scores of TE-derived accessible motifs (the detailed methods are presented in "Sequence conservation in TE-derived cis-elements"). The results showed that the MER130-derived accessible *de novo* motif 1 and the MamRep434-derived accessible *de novo* motif 3 were highly conserved in TE-derived cis-elements among placental mammals.

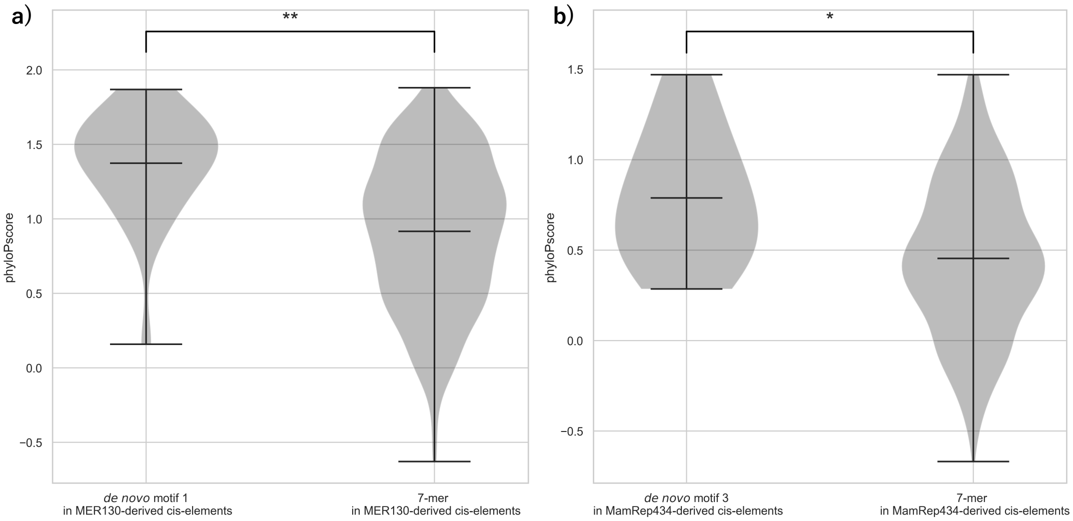

Supplementary Figure 13. *De novo* motifs are highly conserved in TEs. The figure shows the sequence conservation of the accessible motifs within TEs among mammals. The panel (a) shows the conservation of MER130-derived accessible *de novo* motif 1. The panel (b) shows the conservation of the MamRep434-derived accessible *de novo* motif 3. For each panel, the left violin plot (*de novo* motif in TE) shows the sequence conservation score distribution for each accessible motif within the TEs, and the right violin plot (control) shows the sequence conservation score distribution for 500 randomly obtained 7 bp sequences from the intersection of the corresponding TEs and the ATAC peaks. The marks indicate significant differences between the two groups with Mann–Whitney's U test ($*p < 0.05$, $**p < 10^{-5}$).

Comparison of the p300 enrichment MER130 detected by Notwell *et al.* and the MER130-derived cis-element detected in our study.

Supplementary Figure 14 shows the overlap between the TE fragment with p300 enrichment detected by Notwell *et al.* [13], and the TE fragment overlapping with the ATAC peak detected in our study.

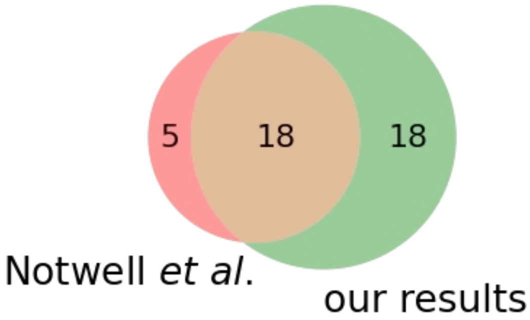

Supplementary Figure 14. A Venn diagram was used to compare the number of the active MER130-derived cis-elements between the result of Notwell *et al.* (detected by p300 enrichment) and that of study (detected by ATAC-seq peaks). Each number shows elements that overlap or do not overlap between the TE fragments with p300 enrichment detected by Notwell *et al.* and the TE fragments overlapping with the ATAC peak as detected in our study. For the comparison, the condition of TE annotation was adjusted to that of the previous method that used nhmmer [14].

NCBI SRA accession numbers used in this study.

The NCBI SRA accession numbers of the sequence data used in this study are shown in Supplementary Table 5.

Supplementary Table 5. NCBI SRA accession numbers used in this study.

Resource or ChIP antigen	accession number
scATAC-seq (prefrontal cortex)	SRX3776097
scRNA-seq	SRP061902
H3K4me1	SRX062940, SRX085445, SRX2357515, SRX2357520, SRX2357525, SRX2357530, SRX5126293, SRX5126294, SRX8339847, SRX8339848, SRX8339849, SRX8339850, SRX8339851, SRX8339852, SRX8339853, SRX8339854, SRX8339855, SRX8339856, SRX8339857, SRX8339858, SRX8339870, SRX8339873, SRX8339876, SRX8339878, SRX8339880, SRX8339883, SRX8339885, SRX8339887, SRX8339890, SRX8339892, SRX8339894, SRX8339896
H3K4me3	SRX062941, SRX081810, SRX081811, SRX085449, SRX1134814, SRX2263643, SRX2263644, SRX2263658, SRX2263659, SRX2357516, SRX2357521, SRX2357526, SRX2357531, SRX2966004, SRX2966006, SRX2966008, SRX2966010, SRX2966012, SRX2966014, SRX2966016, SRX2966018, SRX2966020, SRX2966022, SRX2966024, SRX2966026, SRX3231471, SRX323777, SRX323778, SRX323779, SRX323780, SRX3242084, SRX3242085, SRX3242132, SRX5016621, SRX5016622, SRX5126297, SRX5126298
Neurod2	SRX1979968, SRX1979969, SRX1979970
Lhx2	SRX2487346, SRX2487347, SRX2487356, SRX3064886
input	Neurod2: SRX1979972, Lhx2: SRX2487350, SRX2487351, SRX3064887

References

1. Cusanovich, D. A. *et al.* A Single-Cell Atlas of In Vivo Mammalian Chromatin Accessibility. *Cell* **174,** 1309–1324 (2018).
2. Kulakovskiy, I. V. *et al.* HOCOMOCO: towards a complete collection of transcription factor binding models for human and mouse via large-scale ChIP-Seq analysis. *Nucleic Acids Res* **46,** D252–D259 (2018).
3. Gupta, S., Stamatoyannopoulos, J. A., Bailey, T. L. & Noble, W. S. Quantifying similarity between motifs. *Genome Biol* **8,** R24 (2007).
4. Chen, H. *et al.* Single-cell trajectories reconstruction, exploration and mapping of omics data with STREAM. *Nat Commun* **10,** 1903 (2019).
5. Hodge, R. D., Kahoud, R. J. & Hevner, R. F. Transcriptional control of glutamatergic differentiation during adult neurogenesis. *Cell Mol Life Sci* **69,** 2125–2134 (2012).
6. Chou, S. J. & Tole, S. Lhx2, an evolutionarily conserved, multifunctional regulator of forebrain development. *Brain Res* **1705,** 1–14 (2019).
7. Li, H. *et al.* Transcription factor MEF2C influences neural stem/progenitor cell differentiation and maturation in vivo. *Proc Natl Acad Sci U S A* **105,** 9397–9402 (2008).
8. Roybon, L. *et al.* Neurogenin2 directs granule neuroblast production and amplification while NeuroD1 specifies neuronal fate during hippocampal neurogenesis. *PLoS One* **4,** e4779 (2009).
9. Lim, D. A. & Alvarez-Buylla, A. The Adult Ventricular-Subventricular Zone (V-SVZ) and Olfactory Bulb (OB) Neurogenesis. *Cold Spring Harb Perspect Biol* **8** (2016).
10. Schep, A. N., Wu, B., Buenrostro, J. D. & Greenleaf, W. J. chromVAR: inferring transcription-factor-associated accessibility from single-cell epigenomic data. *Nat Methods* **14,** 975–978 (2017).
11. De Lombares, C. *et al.* Dlx5 and Dlx6 expression in GABAergic neurons controls behavior, metabolism, healthy aging and lifespan. *Aging (Albany NY)* **11,** 6638–6656 (2019).
12. McLean, C. Y. *et al.* GREAT improves functional interpretation of cis-regulatory regions. *Nat Biotechnol* **28,** 495–501 (2010).
13. Notwell, J. H., Chung, T., Heavner, W. & Bejerano, G. A family of transposable elements co-opted into developmental enhancers in the mouse neocortex. *Nat Commun* **6,** 6644 (2015).
14. Wheeler, T. J. & Eddy, S. R. nhmmer: DNA homology search with profile HMMs. *Bioinformatics* **29,** 2487–2489 (2013).
