# Supplementary material for: Transposons contribute to the acquisition of cell type-specific cis-elements in the brain
Source: Commun Biol. 2023 Jun 10;6:631. doi: 10.1038/s42003-023-04989-7 (PMC10257727; doi:10.1038/s42003-023-04989-7)
Supplement: Supplementary file 5 — Reporting Summary [file 42003_2023_4989_MOESM5_ESM.pdf]

nature portfolio

Corresponding author(s): Michiaki Hamada, Masahiro Onoguchi

Last updated by author(s): May 4, 2023

Reporting Summary

Nature Portfolio wishes to improve the reproducibility of the work that we publish. This form provides structure for consistency and transparency in reporting. For further information on Nature Portfolio policies, see our Editorial Policies and the Editorial Policy Checklist.

Statistics

For all statistical analyses, confirm that the following items are present in the figure legend, table legend, main text, or Methods section.

n/a	Confirmed	
☐	☒	The exact sample size (*n*) for each experimental group/condition, given as a discrete number and unit of measurement
☐	☒	A statement on whether measurements were taken from distinct samples or whether the same sample was measured repeatedly
☐	☒	The statistical test(s) used AND whether they are one- or two-sided<br>*Only common tests should be described solely by name; describe more complex techniques in the Methods section.*
☒	☐	A description of all covariates tested
☐	☒	A description of any assumptions or corrections, such as tests of normality and adjustment for multiple comparisons
☐	☒	A full description of the statistical parameters including central tendency (e.g. means) or other basic estimates (e.g. regression coefficient) AND variation (e.g. standard deviation) or associated estimates of uncertainty (e.g. confidence intervals)
☐	☒	For null hypothesis testing, the test statistic (e.g. *F*, *t*, *r*) with confidence intervals, effect sizes, degrees of freedom and *P* value noted<br>*Give P values as exact values whenever suitable.*
☒	☐	For Bayesian analysis, information on the choice of priors and Markov chain Monte Carlo settings
☒	☐	For hierarchical and complex designs, identification of the appropriate level for tests and full reporting of outcomes
☒	☐	Estimates of effect sizes (e.g. Cohen's *d*, Pearson's *r*), indicating how they were calculated

Our web collection on statistics for biologists contains articles on many of the points above.

Software and code

Policy information about availability of computer code

Data collection	No software was used to collect the data.
Data analysis	BEDTools v2.30.0<br>bowtie2<br>chromVAR (Bioconductor version: Release 3.14)<br>FIMO v5.4.1<br>g:Profiler (version e104_eg51_p15_3922dba)<br>GREAT v4.0.4<br>Jalview v2.11.2.6<br>MACS2 v3.7.2<br>Mafft v7<br>Seurat v4.1.0<br>STREAM v1.1<br>TOMTOM v5.4.1<br>UCSC Genome Browser (UCSC LiftOver, PhyloP conservation, RepeatMasker) last updated: Jul. 2007 assembly of the mouse genome (mm9, NCBI Build 37)<br>python3 v3.9.6<br>R v4.1.3

For manuscripts utilizing custom algorithms or software that are central to the research but not yet described in published literature, software must be made available to editors and reviewers. We strongly encourage code deposition in a community repository (e.g. GitHub). See the Nature Portfolio guidelines for submitting code & software for further information.

March 2021

Data

Policy information about availability of data

All manuscripts must include a data availability statement. This statement should provide the following information, where applicable:

- Accession codes, unique identifiers, or web links for publicly available datasets
- A description of any restrictions on data availability
- For clinical datasets or third party data, please ensure that the statement adheres to our policy

The public single-cell ATAC-seq data used in this study have been provived in the GEO Repository with the accession code GSE111586.
The public single-cell RNA-seq data used in this study have been provived in the GEO Repository with the accession code GSE71585.
The public ChIP-seq data used in this study have been provived in ChIP-Atlas (https://chip-atlas.org/). The accession codes used in this study are listed in Supplementary Table 5.

Field-specific reporting

Please select the one below that is the best fit for your research. If you are not sure, read the appropriate sections before making your selection.

☒ Life sciences ☐ Behavioural & social sciences ☐ Ecological, evolutionary & environmental sciences

For a reference copy of the document with all sections, see nature.com/documents/nr-reporting-summary-flat.pdf

Life sciences study design

All studies must disclose on these points even when the disclosure is negative.

Sample size	As we only used public datasets in our study, we used the original sample size from each public dataset.
Data exclusions	We used the single-cell ATAC-seq data of the labels including more than 100 cells (labels were defined in previous published data-sets [Cusanovich, DA et al., Cell, 2018]) to support meaningful conclusions. In addition, in the pseudotime analysis with STREAM, we only used neuron-labelled cells to order the cells along with the neuronal differentiation state (Figure 3).<br>To prevent the enrichment score from being abnormally high due to a small number of TE fragments, we restricted the subfamilies to transposable elements (TEs) that intersected the ChIP peak at least three times in the whole genome. We also restricted the subfamilies where ATAC peaks and motif-containing TEs intersected at least three times in the whole genome (Figure 5).<br>For calculation of TE accessibility, to exclude cases in which TEs were assessed as abnormally accessible, we restricted our analysis to the TE subfamily, in which more than 100 neuron-labeled or CThPN(corticothalamic projection excitatory neurons)-labeled cells overlapped with at least one ATAC peak (Figure 5).
Replication	Not relevant as we did not conduct any wet experiment in our study.
Randomization	Not relevant as we did not conduct any wet experiment in our study.
Blinding	Not relevant as we only used public datasets in our study.

Reporting for specific materials, systems and methods

We require information from authors about some types of materials, experimental systems and methods used in many studies. Here, indicate whether each material, system or method listed is relevant to your study. If you are not sure if a list item applies to your research, read the appropriate section before selecting a response.

Materials & experimental systems

n/a	Involved in the study	
☒	☐	Antibodies
☒	☐	Eukaryotic cell lines
☒	☐	Palaeontology and archaeology
☒	☐	Animals and other organisms
☒	☐	Human research participants
☒	☐	Clinical data
☒	☐	Dual use research of concern

Methods

n/a	Involved in the study	
☒	☐	ChIP-seq
☒	☐	Flow cytometry
☒	☐	MRI-based neuroimaging
